# Determination of Strain Limits for Dimensioning Polyurethane Components

**DOI:** 10.3390/polym13183198

**Published:** 2021-09-21

**Authors:** Michael Stanko, Peter Lehmenkühler, Markus Stommel

**Affiliations:** 1 Chair of Plastics Technology, TU Dortmund University, Leonhard-Euler-Str. 5, D-44227 Dortmund, Germany; peter.lehmenkuehler@tu-dortmund.de; 2 Leibniz Institute of Polymer Research, Hohe Str. 6, D-01069 Dresden, Germany; stommel@ipfdd.de

**Keywords:** strain limit, polyurethane, lightweight engineering, residual energy ratio

## Abstract

Within the scope of this contribution, a method for the determination of a strain limit for designing components made of elastomeric polyurethane systems is presented. The knowledge of a material-specific strain limit is essential for the structural-mechanical calculation of plastic components in the context of component design. Compared to a commonly used component design, based on a simplified dimensioning approach taking only linear viscoelastic deformations into account, the strain limit determined in this study allows an improved utilisation of lightweight construction potential in the dimensioning of technical components made of polyurethanes through the consideration of permissible nonlinear viscoelastic deformations. The test method comprises a sequence of quasi-static loading and unloading cycles, with a subsequent load-free recovery phase, allowing the relaxation of the viscoelastic forces. Standardised tensile and simple shear test specimens and a dynamic mechanical thermal analyser (DMTA) are used within the tests. The strain limit is determined by means of the so-called residual energy ratio, which is a characteristic quantity for the evaluation of hystereses of load–unload cycles. These hystereses are increasingly formed by deformations outside the range of linear viscoelastic deformations. The residual energy ratio relates the proportion of deformation energy recovered during unloading to the deformation work that is applied. In this contribution, the residual energy ratio is successfully used to detect a significant evolution of loss energy under increasing load and to correlate this transition to a characteristic strain. The latter is used as a dimensioning parameter for the design of components made of elastomeric polyurethane materials for quasi-static load cases. The determination of this strain limit is performed under consideration of the criterion of reversibility of deformation.

## 1. Introduction

Depending on their chemical composition, the mechanical properties of polyurethanes (PUR) include the entire range of material behaviour of engineering plastics, from rubber-elastic to hard-elastic brittle deformation behaviour. The characterisation and modelling of the material behaviour and the determination of mechanical parameters of elastomeric polyurethanes are of high importance for engineering applications. Furthermore, only a few data sets with basic material properties are available in the literature, such as those used for thermoplastics in the form of the database CAMPUS, for example. This also results in a deficit regarding appropriate design parameters for polyurethane materials for the performance of strength assessments as they are known for thermoplastics or composite materials. Basically, the design methods for plastic components with corresponding design parameters and their limit values are divided into stress-, strain-, and energy-based approaches. The advantages of a strain-based design using a so-called critical strain or limit strain are presented, for example, in investigations of Menges [1,2] and Kunz [3,4]. For more detailed information on common dimensioning criteria, design parameters, and associated limit values, reference is made to [5,6].

In the simplified case, carrying out a strain-based strength analysis, a strength is defined as a permissible strain $\varepsilon_{per}$ and compared with a locally calculated strain by means of an equivalent strain $\varepsilon_{eq\;max}$. (for example, from a finite element analysis—FEA):

(1) $$\varepsilon_{eq\;max}\;\leq \;\varepsilon_{per}=\varepsilon_{lim}\cdot \frac{K}{S}$$

The permissible strain $\varepsilon_{per}$ is determined by reducing a material characteristic limit strain $\varepsilon_{lim}$ by a safety factor S, and can also be adjusted by application-specific influence factors K to take into account, for example, environmental influences. The strain limit $\varepsilon_{lim}$ is determined from stress–strain curves, considering the viscoelastic properties of the analysed polymer material (time and temperature dependency).

In studies such as [7,8,9,10], the characterisation and modelling of the viscoelastic properties of elastomeric (non-foamed) polyurethanes are presented, but no information on dimensioning parameters for a component design is given. However, a material model that accurately describes the real deformation behaviour of a polyurethane component is not sufficient for an engineer to carry out a mathematical evaluation of whether a component can withstand the applied loads or whether its material will be damaged due to the resulting stresses. Furthermore, in many cases, the stress–strain curves can only be described accurately by means of a material model up to a defined strain. Therefore, from the engineer’s point of view, the material-specific design limits also determine the technically significant range of strain, which has to be adequately represented by the model.

**Figure 1:**
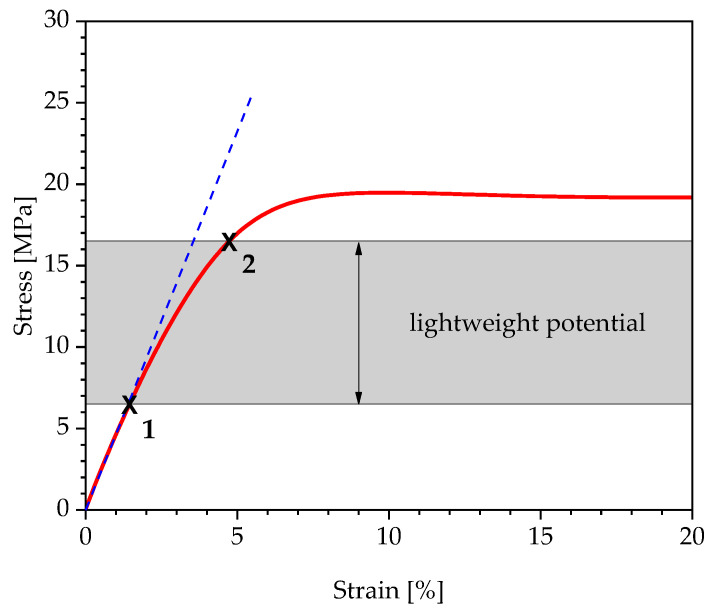
Schematic illustration of the utilisation of lightweight construction potential by considering reversible nonlinear viscoelastic deformation components.

Studies such as [11], which provide comprehensive overviews of the essential characteristic values for the design of plastic components, contain only general information regarding the mechanical parameters and stress-based design values for polyurethanes. In the absence of the knowledge about design limitations, in the following, a so-called simplified design assumption is often carried out in practice, as shown in Figure 1, using the example of a stress–strain curve of a uniaxial tensile test. In this context, only linear viscoelastic deformations are evaluated as permissible, and permissible maximum strains are determined at the proportionality threshold of the stress–strain curve (see Figure 1, point no.1). The essential disadvantage of a simplified design is the neglect of the reversible nonlinear viscoelastic deformation components and, thus, the utilisation of additional lightweight design potential (Figure 1, point no.2).

Due to the wide range of commercially available polyurethane systems and the associated spectrum of mechanical properties, there is also a requirement for a universal test method for the determination of design limits outside the linear viscoelastic deformation range. The design criterion considered in this study is based on the condition of reversibility of deformation [12,13]. The design limit is not the proportionality limit of a stress–strain curve, but a strain limit, defined in the range of nonlinear reversible strains before irreversible deformations of the material can be determined. This transition can be detected by means of increasing-load tests, which include corresponding relaxation phases between the individual loading cycles. Approaches such as these are taken up, for example, in [14,15] to determine the transition between reversible and irreversible deformation processes in thermoplastics. The method of the authors provides for the performance of a loading and unloading process, with subsequent relaxation of the restoring forces over a period of 300 s (empirically determined period for the purpose of relaxation of the viscoelastic forces) and increasing the load level by a defined strain increment. Using the representation of the residual strain at the end of the restoring phase as a function of the introduced strain of the corresponding load step, the transition to plastic irreversible deformations of the material is determined. For polyurethanes, similar increasing-load tests with subsequent relaxation phases have already been carried out by Goldschmidt and Diebels [16] and Johlitz et al. [17] to characterise and model the viscoelastic material behaviour. However, the definition of a design limit for the component design is not considered.

## 2. Materials and Methods

### 2.1. Materials

**Figure 2:**
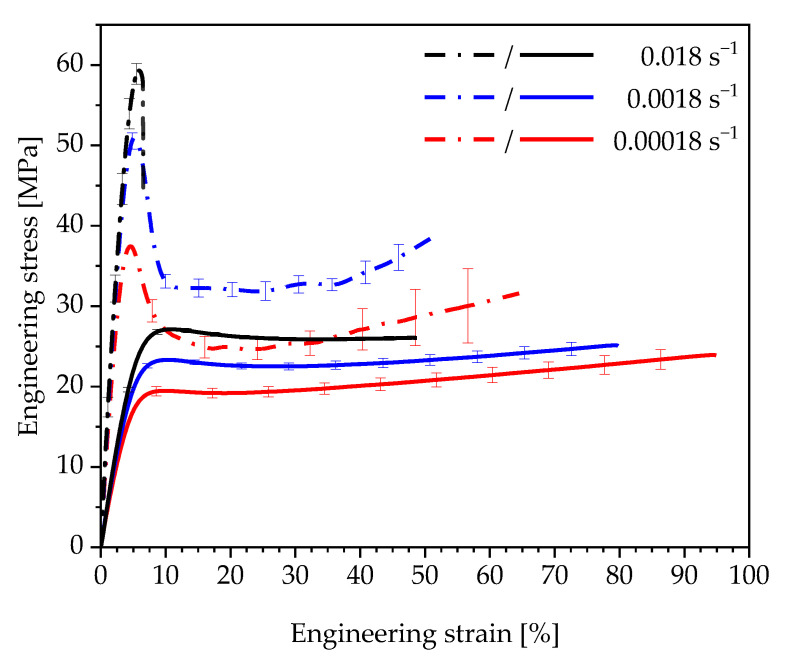
Strain-rate-dependent stress–strain curves of Neukadur^®^ (—) and puroclear^®^ (- · -) under uniaxial tensile load (type 1A test specimens according to DIN EN ISO 527-2).

The experimental investigations are carried out on two elastomeric polyurethane systems: Neukadur^®^ PN 9740 (Altropol Kunststoff GmbH, Stockelsdorf, Germany) and puroclear^®^ 3351/1 IT (Rühl Puromer GmbH, Friedrichsdorf, Germany). Both polyurethane types possess a strain-rate-dependent viscoelastic material behaviour but differ basically in terms of stiffness and the stress–strain curve (Figure 2).

The uniaxial material behaviour of both materials is comparable to that of other elastomeric polyurethanes [8,18,19]. The stress–strain curves are characterised by an initially linear viscoelastic deformation behaviour. After reaching a specific degree of stretching, the stress increases nonlinearly until the material fails.

### 2.2. Methods

The test method for the determination of a dimensioning limit for polyurethanes presented in this contribution comprises a sequence of cyclic loading and unloading procedures, with subsequent load-free relaxation phases and a continuous load increase. The test approach is based on the dimensioning criterion of reversibility of the deformation. However, deformation processes up into the nonlinear viscoelastic range are considered, which lead to the formation of hystereses due to energy dissipation during deformation.

**Figure 3:**
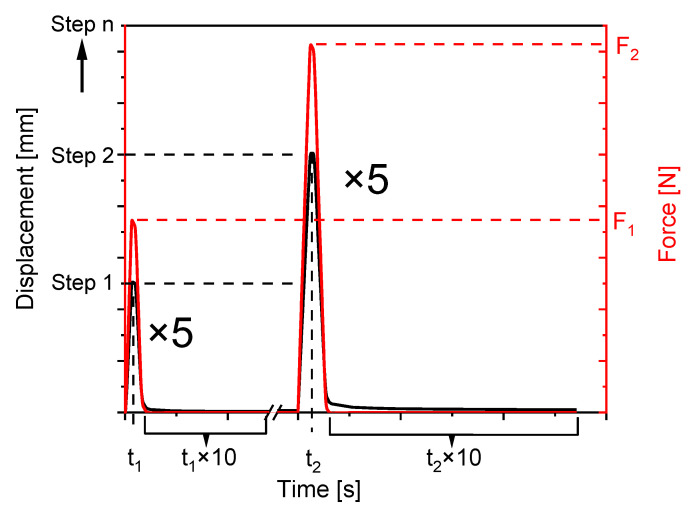
Test sequence of the increasing-load test to determine a strain limit for polyurethanes.

Standardised tensile test specimens of type 1A according to DIN EN ISO 527-2 are used in the increasing-load tests. The mechanical testing is performed with a DMTA machine of the type E10000 (Instron GmbH, Darmstadt, Germany). The measuring procedure consists of five consecutive load cycles for 14 different load levels (strain amplitudes). The specimen is first deformed to 0.5 mm, unloaded, and kept free of force for a defined time interval. The latter aims for the relaxation of viscoelastic forces of the loaded and unloaded specimen (Figure 3).

**Figure 4:**
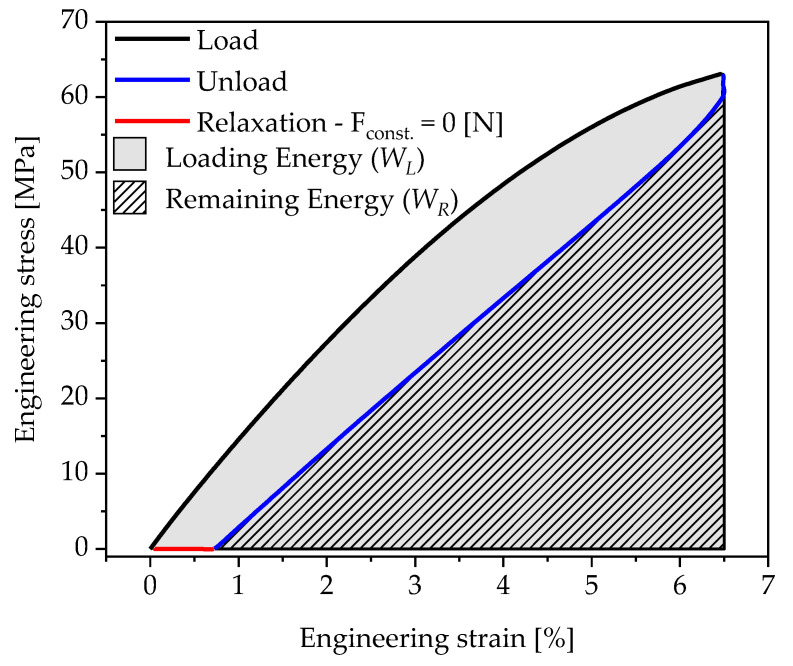
Stress–strain curve of a test cycle with loading, unloading, and relaxation phase.

The time interval for relaxation is empirically determined for both materials in preliminary tests and amounts 10 times of the load time for the respective load level. It is important to ensure that the viscoelastic forces are removed as completely as possible, considering a practicable total test duration (the sum of all load cycles with the respective repetitions). The total test duration increases with the reduction of the loading and unloading speed, as well as with each further load level considered. The measuring procedure of the DMTA is defined in the way that, during a relaxation phase, the specimen is guided load-free (F = 0 N) to the initial length of the specimen (Figure 4). The return to the initial length is, thus, completely detected by the testing machine and does not have to be determined in a further step after the specimen has been unclamped. After five repetitions of a load step, the displacement is increased by 0.5 mm and the relaxation phase is adjusted by 10 times of the resulting load time.

By integrating the engineering stress $\sigma_{E}$ over the engineering strain $\varepsilon_{E}$, the loading energy density of a loading procedure is determined:

(2) $$W=\int \sigma_{E}\;d\varepsilon_{E}$$

For a given load cycle, the loading energy density $W_{L}$ applied during loading, as well as the remaining energy density $W_{R}$, can be determined. The loading energy density $W_{L}$ is reduced by the amount of dissipated energy and released during unloading (Figure 4). As in [20,21], the energy loss is quantified using the so-called residual energy ratio $\phi_{Res}$ that correlates with the amount of dissipated energy [22]. It is defined by the ratio of the energy loss ($W_{L}-W_{R}$) to the loading energy density $W_{L}$ applied:

(3) $$\phi_{Res}=\frac{W_{L}-W_{R}}{W_{L}}=\frac{\int \sigma_{Load}\;d\varepsilon_{Load}-\int \sigma_{Unload}\;d\varepsilon_{Unload}}{\int \sigma_{Load}\;d\varepsilon_{Load}}$$

**Figure 5:**
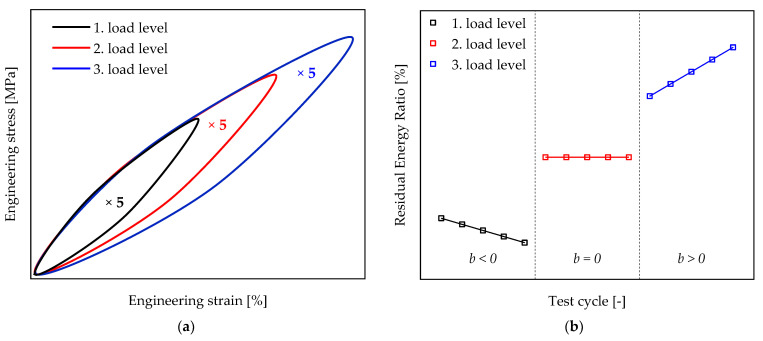
Determination of the residual energy ratio within a load level: (**a**) schematic hysteresis curves of different load levels; (**b**) schematic approximation of the determined residual energy ratio of a load level by straight lines.

For each repetition of a load level (Figure 5a, five repetitions), the residual energy ratio is calculated according to Equation (3). For each load level, this results in five successive measurement points of the residual energy ratio. All measurement points are then plotted over the corresponding measurement cycle number or cycle time. The paths of the residual energy ratio within a load level are approximated by a line using the five measuring points (Figure 5b).

If stresses within the repetitions of a load level are reduced by relaxation processes in the material, the residual energy ratio within a cycle will also decrease. If, on the other hand, the residual energy ratio increases with the number of cycles, the amount of energy loss will increase. This is caused by an increasing influence of dissipative effects inside the material. Detailed information regarding this topic can be found in [23,24]. By observing the slope b of the respective straight line of a series of measurements (load step), an analytical distinction is made between a reduction in the residual energy ratio and an increase in this variable (Table 1).

The load level, which is associated with the transition from a dominating cyclic relaxation b<0 to an increasing dissipation effect within the material b>0, is used in this study to define a strain limit. Accordingly, the state is determined in which the slope of the residual energy ratio of a load level meets the condition  b=0, i.e., the residual energy ratio of a load level remains constant ($\phi_{Res}=const.$).

At this point, it must be mentioned that an increasing heating of elastomeric materials during deformation does not necessarily lead to irreversible deformation or damage [25,26,27]. The latter will not be considered more closely in the context of this research. The focus is on the determination of a strain limit outside the linear viscoelastic range, which meets the criterion of reversibility of the deformation and represents an alternative to the simplified dimensioning approach taking into account only linear viscoelastic deformations.

To consider the strain rate dependence of the strain limit to be determined, the measurements are carried out at two different test speeds of 10 mm/min and 100 mm/min. For both test speeds, 10 times the loading time within a cycle is considered as the relaxation time (removal of restoring forces).

### 2.3. Validation of the Test Method

A part of the deformation energy is dissipated to the environment in the form of heat [18]. Depending on the material-specific density ρ and the specific heat capacity c, this results in a temperature change $\dot{T}$ according to the following equation [19]:

(4) $$\dot{T}=\frac{\dot{W_{T}}}{\rho c}$$

**Figure 6:**
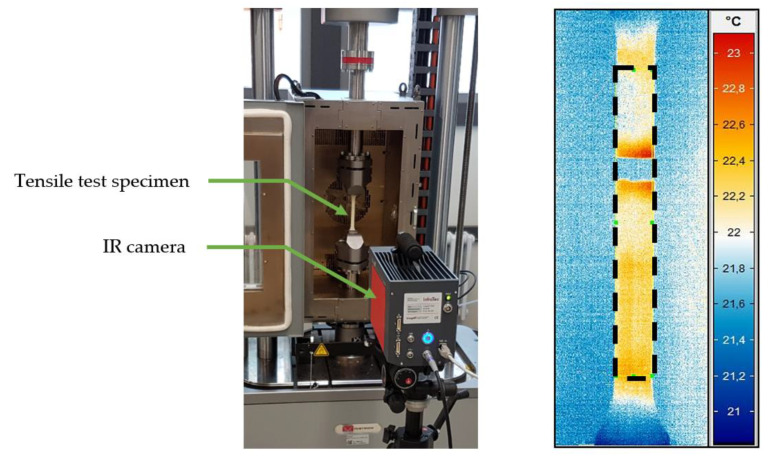
Experimental setup with thermographic measurement for detection of specimen heating.

As described in [28,29,30], the heating of the test specimen can be detected by means of thermographic analyses and considered in connection with the evaluation of viscoelastic effects or damage processes (under progressive load). For example, in [26,27,31], the temperature change in a composite material is measured by means of an infrared (IR) camera system and used to define a dimensioning limit for cyclically tensile stressed test specimens. Based on the results of these studies, the test method is validated within the scope of this contribution by means of an IR camera of the type ImageIR 8300 (InfraTec GmbH, Dresden, Germany), which is used to detect the heating of the tensile specimen in the DMTA during the mechanical test (Figure 6).

The cycle-dependent change in the sample temperature, resulting from the dissipation energy of the deformation process released in the form of heat, is observed here. The correlation of the energy dissipation implied by the increase in the residual energy ratio, with the dissipation determined by thermography during a loading and unloading cycle, is investigated.

### 2.4. Evaluation of the Determined Strain Limits under Simple Shear Load

In addition to the validation of the test methodology, the applicability of this to other load cases is also investigated. In this context, it is evaluated whether a reversible deformation of the material is also given for shear loads up to the strain limit determined according to Section 2.2 under tensile load. A cyclic simple shear test using the Iosipescu test specimen according to ASTM D5379 is used for the investigations. The applied test procedure is similar to that of the cyclic tensile tests (Figure 3 and Figure 4) with loading and unloading phases, as well as the relaxation phase to remove viscoelastic forces. The load increase is carried out with a displacement increment of 0.35 mm up to a displacement of 5 mm. Each load step is repeated five times. In order to compare the obtained strain limits of the cyclic tensile test and the cyclic simple shear test, the respective limit principal strains of the tests are analysed. Principal strains are used in strain-based strength analysis for plastic component design in accordance with the maximum principal strain theory (Equation (5)) [3,32].

(5) $$\varepsilon_{eq\;max}=max\;(\varepsilon_{1},\varepsilon_{2},\varepsilon_{3})\;\leq \;\varepsilon_{per}=\varepsilon_{lim}\cdot \frac{K}{S}$$

**Figure 7:**
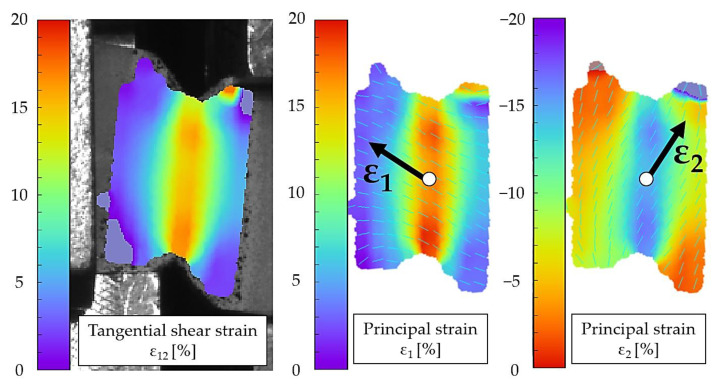
Contour plots of the tangential shear strain and resulting principal strains in the simple shear test for Neukadur^®^ and a displacement of 5 mm.

When a critical strain is reached, the failure of the material is expected. This critical strain or limit strain is compared to a locally prevailing maximum principal strain (e.g., from an FEA). In the case of the uniaxial tensile test, the principal strain is aligned in the loading direction. The determination by means of digital image correlation (DIC) and the orientation of the principal strains $\varepsilon_{1}$ and $\varepsilon_{2}$ within the cyclic simple shear test is shown exemplarily in Figure 7 for a displacement of 5 mm (for the polyurethane resin Neukadur^®^).

Figure 7 shows that the tangential shear strain corresponds to the absolute values of the principal strain $\varepsilon_{1}$ and $\varepsilon_{2}$ in the principal axis system. It is important to note that the test speeds of both tests (tensile and shear test) must be adjusted in such a way that the strain rate of the maximum principal strain in the simple shear test corresponds to that of the tensile test. For the evaluation, a simple shear test equivalent to a tensile test with a test speed of 10 mm/min is considered (${\dot{\varepsilon }}_{1}=$ 0.0018 s^−1^).

## 3. Results and Discussion

### 3.1. Determination of Strain Limit

**Figure 8:**
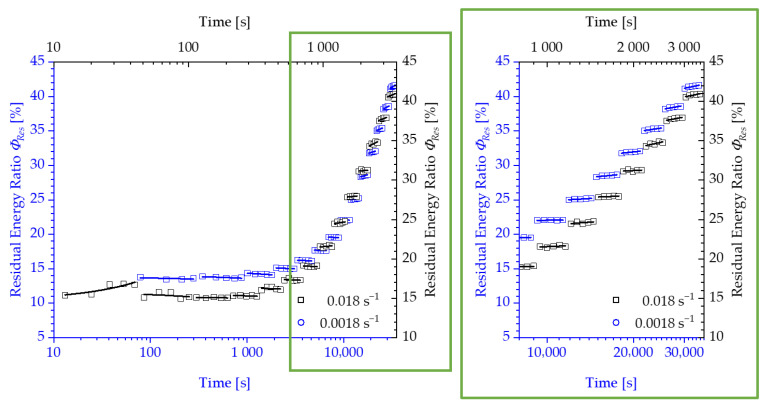
Development of the residual energy ratio $\phi_{Res}$ for Neukadur^®^ for a test speed of 10 mm/s and 100 mm/s.

**Figure 9:**
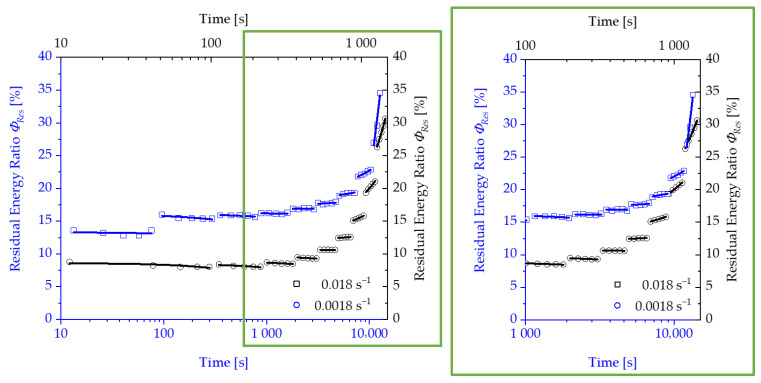
Development of the residual energy ratio $\phi_{Res}$ for puroclear^®^ for a test speed of 10 mm/s and 100 mm/s.

Following the explanations in the previous section, the results of the tests and the determination of strain limits for both considered polyurethane systems are presented below. The change in the residual energy ratio $\phi_{Res}$ as a function of the measurement cycle time is shown in Figure 8 and Figure 9. Due to the relaxation phases between the loading and unloading of the specimen, which are increased by a factor of 10 of the loading time with each subsequent load level, the results are presented using a logarithmic scale of the time.

**Figure 10:**
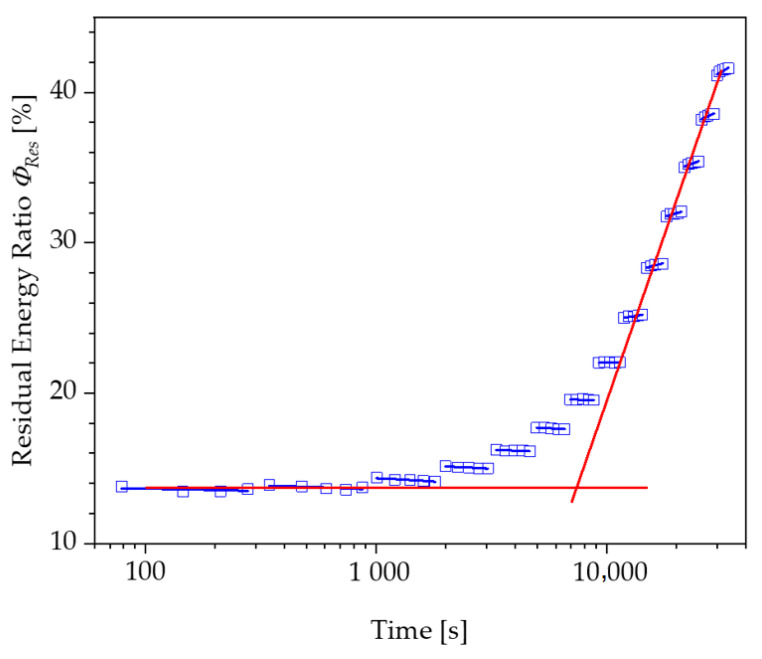
Tangent approach for the determination of a strain limit based on the residual energy ratio curve of the PUR Neukadur^®^.

For both test speeds, a significant increase in the energy loss with increasing loads is recorded after passing through a transition point. Correspondingly, the residual energy ratio $\phi_{Res}$ also increases from a specific load level within the repetitions of a load level. The progression of the residual energy ratio $\phi_{Res}$ above the logarithmically scaled time shows two areas, which can be approximated by two tangents, as shown in Figure 10.

**Figure 11:**
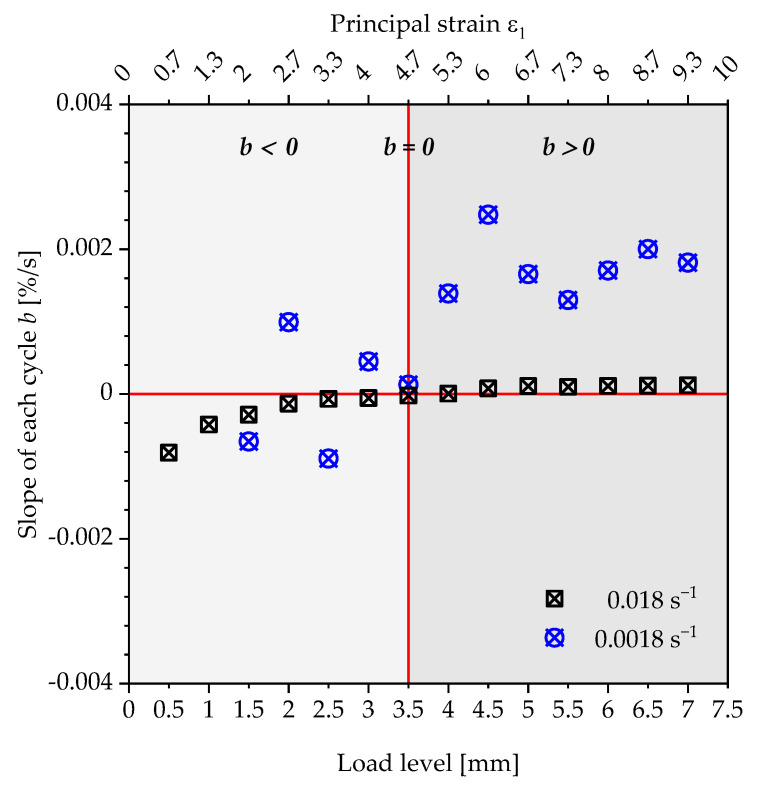
Plot of the slopes of the residual energy ratio curve within the specific load levels respective strain levels for the polyurethane system Neukadur^®^ under tensile load.

**Figure 12:**
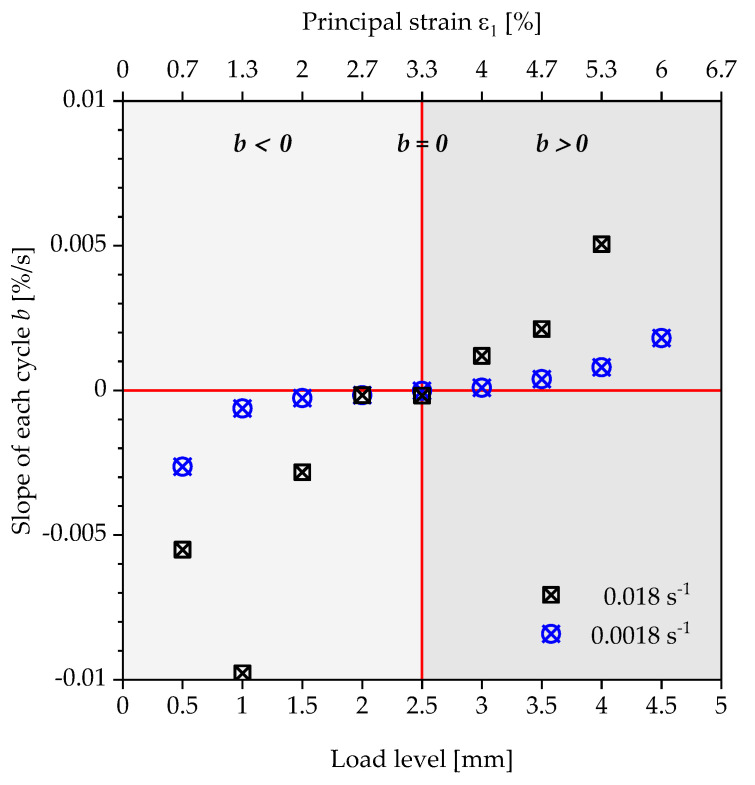
Plot of the slopes of the residual energy ratio curve within the specific load levels’ respective strain levels for the polyurethane system puroclear^®^ under tensile load.

The point of intersection of these tangents characterises the transition with a corresponding load amplitude, above which a steady increase in the energy loss of a loading and unloading cycle occurs. However, the authors would like to discuss an alternative proposal for the determination of the strain limits compared to the evaluation using tangents. Instead of a user-dependent construction of two tangents, the slope of the linear course of the residual energy ratio of a load level is plotted over the displacement of the specimen or the corresponding strain to be assigned to this load (Figure 11 and Figure 12).

Basically, two areas of progression can be observed. In the first area (light grey), the slopes of the straight lines show negative values (b<0), which are caused by the reduction in the energy loss and the cyclic relaxation during the repetition of a load step. Above a specific load level (dark grey), the slopes of the lines are entirely positive (b>0). The residual energy ratio $\phi_{Res}$ increases with each repetition of a load level, as well as with each load step. Even if cyclic relaxation occurs for higher load steps, the viscoelastic effect becomes the dominant mechanism regarding the change in the residual energy ratio $\phi_{Res}$. These two areas are separated by a state in which the slope of the course of the residual energy ratio $\phi_{Res}$ reaches the value  b=0. The corresponding load level and the resulting strain is defined in this contribution as the strain limit for the quasi-static component design. This can be seen in Figure 12 for the polyurethane of the type puroclear^®^, whereas the course of the polyurethane Neukadur^®^ shows two deviations for the measurement with a strain rate of 0.0018 s^−1^. The latter are considered as outliers, especially since, also for this test series, only positive slopes of the linear path can be observed from a displacement of the test specimen of 3.5 mm. Applying the method described above, the limit values shown in Table 2 are obtained for the condition  b=0.

The strain limit can be assumed as strain-rate-independent for the investigated PUR systems and strain rates. Based on the specimen geometry, the strain limits for puroclear^®^ and Neukadur^®^ result in $\varepsilon_{1\;lim}\;$= 3.3% (load step: 2.5 mm) and $\varepsilon_{1\;lim}\;$= 4.7% (load step: 3.5 mm).

### 3.2. Validation of the Test Method

**Figure 13:**
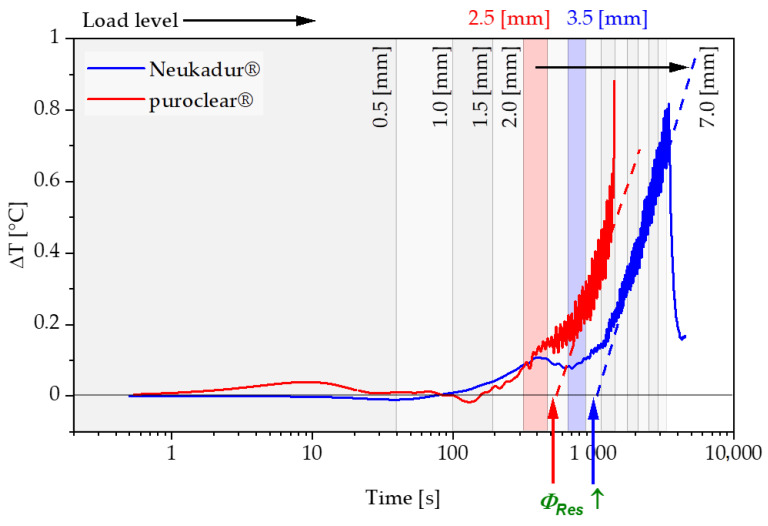
Sample heating during the test procedure for Neukadur^®^ and puroclear^®^ for a strain rate of 0.018 s^−1^ under tensile load.

To validate the measurement methodology presented, the mechanical investigation is combined with thermographic analyses. According to Equation (4), a part of the dissipated energy is released to the environment in the form of heat via the sample surface. The sample temperature increases with increasing load level, respectively, above the strain limit determined in this case with each measuring cycle of a load level, and is determined by means of an IR camera. Figure 13 shows the sample heating of the Neukadur^®^ and the puroclear^®^ system during the test procedure.

The temperature is evaluated within the surface marked in Figure 6. The maximum value of the sample temperature in the measuring region of the tensile test specimen is considered for the validation. The temperature curves versus time exhibit a bilinear characteristic in logarithmic representation and can be approximated by two tangents. The intersection point of both tangents defines the extrapolated beginning of a significant heat dissipation increase. Considering the load steps at these time points and comparing them with the limit values determined from Figure 11 and Figure 12 for the state b=0, a conformity can be observed (Table 3).

The results of the thermographic analysis and the determined heat transfer into the environment, thus, confirm the plausibility of the determined parameters, which were obtained by means of the curves of the residual energy ratio.

### 3.3. Evaluation of the Determined Strain Limits under Simple Shear Load

**Figure 14:**
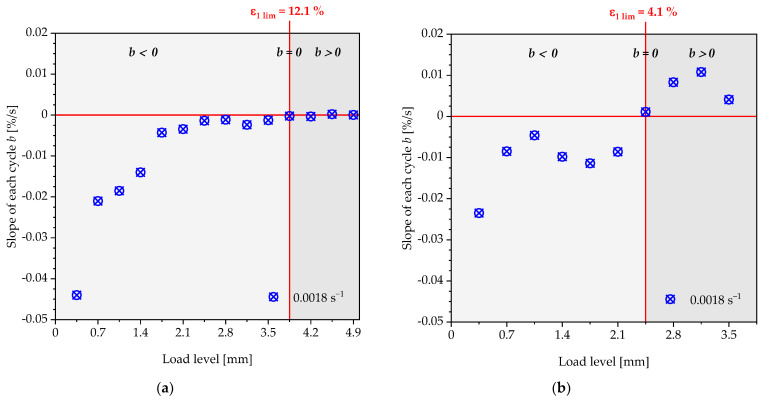
Plot of the slopes of the residual energy ratio curve within the specific load levels’ respective strain levels for the polyurethane systems (**a**) Neukadur^®^ and (**b**) puroclear^®^ under simple shear load.

The results of the application of the test method to the simple shear load case are shown in Figure 14 as plots of the residual energy ratio for the polyurethane systems Neukadur^®^ and puroclear^®^ and a strain rate of 0.0018 s^−1^.

As in Section 3.1, a strain is determined for the considered tests at which the slope of the change in the residual energy ratio within the repetitions of a load level meets the condition  b=0. For the Neukadur^®^, a continuous increase in the slope can be observed up to the load level of 3.85 mm. From this load level onwards, an increase of the residual energy coefficient due to a positive slope b can be noted. For the puroclear^®^, the slope ranges with b<0 and b>0 can be separated clearly. The deformation mechanism changes from cyclic relaxation to increasing viscoelastic effects are present here at a load level of 2.45 mm. For the puroclear^®^, the analysis ends at a load level of 3.85, as, above this level, the specimen fails. Table 4 compares the resulting strain limits $\varepsilon_{1\;lim}$ under consideration of the principal strain $\varepsilon_{1}$ for the tensile test and the simple shear test.

It can be observed that the strain limits determined in the cyclic simple shear test are higher than those obtained under tensile loading. Up to the strain limits of 4.7% (Neukadur^®^) and 3.3% (puroclear^®^) determined under tensile load, a decrease in the residual energy ratio (b<0) can be observed in the cyclic simple shear test. The deformation is, therefore, reversible and is associated with cyclic relaxation within the repetition of a load level. Basically, this means that, according to the tests considered and the validation of the test method, it can be assumed that the dimensioning values under tensile loads are appropriate. Compared to the simple shear test, these exhibit a degree of safety.

The differences in the obtained strain limits can be attributed to technical aspects of the test setup, as well as to mechanical issues. Ziółkowski [33], for example, highlights that the simple shear test is a deformation process that depicts a response to a rotating principal axis system of strain. The simple shear test should, therefore, also be compared with the pure shear test to evaluate the influence of the morphology on the mechanical behaviour of the material. In the latter, the principal axis system remains fixed in its orientation throughout the entire test. Thus, it must be verified whether the results of a characterisation or parameter identification are influenced by this circumstance. Furthermore, Ziolkowski notes that it is technically not possible to realise a homogeneous strain distribution in the simple shear test, as this is influenced by an occurring bending moment and the resulting normal forces. This effect is amplified by the fact that test setups for realising a simple shear test are often carried out in such a way that a displacement of the clamps orthogonal to the loading direction and away from each other is possible with increasing deformation of the test specimen [34].

**Figure 15:**
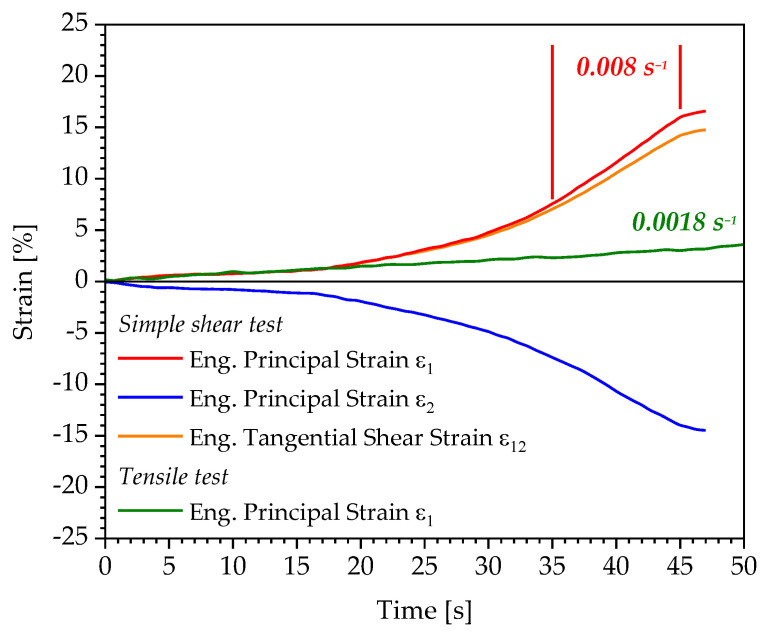
Time-dependent curves of the strains within a quasi-static simple shear and tensile test on the example of the polyurethane system Neukadur^®^.

The influences of these disturbance factors on the simple shear test (quasi-static test up to a specimen displacement of 5 mm) are exemplarily shown in Figure 15 for the time-dependent strain curves of the principal strains and tangential shear strain. These are compared to the strain curve of an equivalent tensile test with a strain rate of 0.0018 s^−1^.

The illustration indicates that the strain rate of the principal strain $\varepsilon_{1}$ in the shear test initially corresponds to that of the equivalent tensile test with a value of 0.0018 s^−1^. Due to the influences described above and the superimposition of the deformation components on those of the simple shear test, the strain rate increases significantly up to the end of the test at a displacement of 5 mm and reaches the value of 0.008 s^−1^. This circumstance causes the significantly larger deviation in the strain limit determined under shear load compared to the strain limit obtained under tensile load for the polyurethane system Neukadur^®^ (Table 4). The higher the load level at which the condition b=0 and, thus, a strain limit is detected in the increasing-load simple shear test, the greater the influence of occurring normal forces on the tangential shear strain or principal strain obtained. Therefore, it is necessary to verify whether the pure shear test represents a more adequate load condition for the verification and extension of the findings on the determined strain limits for the considered polyurethane systems in this contribution.

### 3.4. Evaluation of the Determined Strain Limits in the Context of Increasing the Lightweight Design Potential

**Figure 16:**
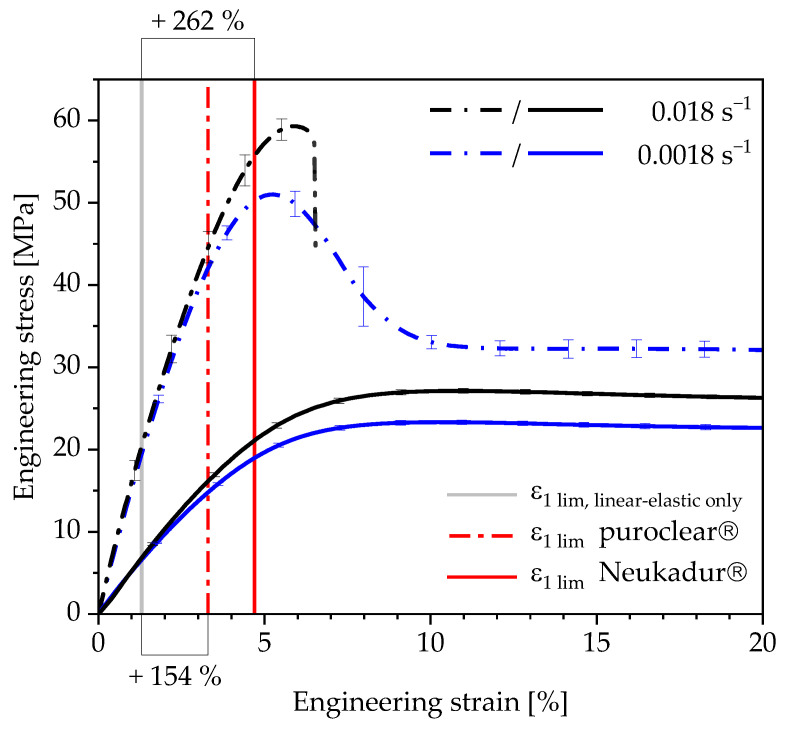
Determined strain limits for the polyurethane systems Neukadur^®^ and puroclear^®^ depicted on the related stress–strain curves.

In Figure 16, the strain limits determined for the considered polyurethane systems are related to the corresponding stress–strain curves. For the puroclear^®^, a 154% higher strain can be applied for the component design. In the case of the Neukadur^®^, the strain limit is 262% higher than that of a simplified design taking only reversible linear viscoelastic deformations into account. 

Especially for the future design of parts including these polyurethane systems, the material behaviour and the permissible material stress is known better. This leads to a better exploit of lightweight potential. During the development of parts containing these polyurethanes, the determined strain limits can be considered in structural-mechanical calculations. Taking these limits into account, the parts can be designed to be more lightweight regarding the simplified design method.

## 4. Conclusions

Within the scope of this contribution, a test method for the determination of a design parameter in the form of a strain limit for the strain-based design of components made of elastomeric polyurethanes is presented. In contrast to a designing method taking linear viscoelastic deformations into account, the determined strain limits contain reversible nonlinear viscoelastic deformation and allow an additional utilisation of the lightweight design potential for polyurethane materials by an increased material exploitation. A cyclic increasing-load tensile test is used for this purpose. Within five successive load cycles, the change in the residual energy ratio is evaluated, and the energies dissipated in the form of heat are quantified. A characteristic significant increase in the residual energy ratio occurred during the test, and the associated load level is used to define the strain limit. The validation of the method is carried out using thermographic analyses and the detection of the sample temperature change during the experiment. These confirm the relationship between the increase in loss energy, implied by the change in the residual energy ratio, and the increase in loss energy due to heat transfer from the sample to the environment. Therefore, the determination of a strain limit according to the principle shown above can be carried out in a purely mechanical test without further measuring equipment, such as thermography. The measuring method can also be carried out with tensile testing machines, which enable cyclical testing procedures. In addition, the applicability of this test method to other load cases is demonstrated in first increasing-load simple shear tests. The equivalent strain limits (principal strain $\varepsilon_{1}$) determined under a uniaxial tensile load are confirmed in this context as safe dimensioning values. In addition, deviations concerning the strain rate in the simple shear test are presented, which lead to the necessity of reviewing the test method and the determined limit values in the pure shear test.

Generally, in further investigations, the test method for uniaxial tensile load is to be extended to further load cases, such as bi- and multi-axial load cases. The investigations for determination of a strain limit under shear load are to be extended to a broader range than shown exemplarily here within the validation for a single strain rate. A more fine-step increasing-load test (smaller displacement or strain increments close to the change in slope b respective to deformation mechanism change) should enable an even more precise definition of the strain limit, and is also to be taken up in future research activities. To confirm the general validity of this test method for the material class of elastomeric plastics, similar tests should be carried out for other polymer materials.

Further development potential of the test method presented exists in the separation of reversible nonlinear viscoelastic deformations above the strain limits determined in this study from reversible deformations that lead to damage of the material. Above the determined strain limit, the specimen heating increases, but this does not necessarily lead to a damage of the material structure. By separating these two areas, the strain limit relevant for the component design could be shifted to larger permissible strains. A further increase in the lightweight design potential would, thus, be achieved.

## Figures and Tables

**Table 1:** Relationship between the slope b and the residual energy ratio $\phi_{Res}$.

Slope *b*	Residual Energy Ratio $\phi_{Res}$	Deformation Mechanism
*b* < 0	$\phi_{Res}↓$	cyclic stress relaxation
*b* = 0	$\phi_{Res}=const.$	change of dominating mechanism
*b* > 0	$\phi_{Res}↑$	increasing energy dissipation

**Table 2:** Strain limits $\varepsilon_{lim}\;$ determined using the mechanical test method for Neukadur^®^ and puroclear^®^ under tensile load.

PUR	Strain Rate (s^−1^)	Load Level (mm)	Tensile Strain Limit $\varepsilon_{lim}\;$ (%)
Neukadur^®^	0.018	3.5	4.7
	0.0018	3.5	4.7
puroclear^®^	0.018	2.5	3.3
	0.0018	2.5	3.3

**Table 3:** Strain limits $\varepsilon_{lim}\;$ determined using the thermographic analysis for Neukadur^®^ and puroclear^®^ under tensile load.

PUR	Strain Rate (s^−1^)	Time _load level_ (s)	Load Level (mm)	Tensile Strain Limit $\varepsilon_{lim}\;$ (%)
Neukadur^®^	0.018	993	3.5	4.7
	0.0018	7979	3.5	4.7
puroclear^®^	0.018	510	2.5	3.3
	0.0018	5105	2.5	3.3

**Table 4:** Strain limits $\varepsilon_{1\;lim}$ determined using the mechanical test method for Neukadur^®^ and puroclear^®^ under tensile and simple shear load.

PUR	Load Level (mm)		Strain Limit $\varepsilon_{1\;lim}$ (%)	
	Tensile Load	Shear Load	Tensile Load	Shear Load
Neukadur^®^	3.5	3.85	4.7	12.1
puroclear^®^	2.5	2.45	3.3	4.1

## Data Availability

The data presented in this study are available on request from the corresponding author.
